# Systematic analysis of human oncogenic viruses in colon cancer revealed EBV latency in lymphoid infiltrates

**DOI:** 10.1186/1750-9378-9-18

**Published:** 2014-06-03

**Authors:** Loretta Fiorina, Mattia Ricotti, Alessandro Vanoli, Ombretta Luinetti, Elena Dallera, Roberta Riboni, Stefania Paolucci, Silvia Brugnatelli, Marco Paulli, Paolo Pedrazzoli, Fausto Baldanti, Vittorio Perfetti

**Affiliations:** 1 Department of Onco-Hematology, Oncology Section, Fondazione IRCCS Policlinico San Matteo, V.le Camillo Golgi 19, 27100 Pavia, Italy; 2 Department of Diagnostic Medicine, Molecular Virology, Fondazione IRCCS Policlinico San Matteo, Pavia, Italy; 3 Department of Molecular Medicine, University of Pavia and Anatomic Pathology section, Fondazione IRCCS Policlinico San Matteo, Pavia, Italy

**Keywords:** Colon cancer, Oncogenic viruses, EBV

## Abstract

**Background:**

Environmental factors may play a role in colon cancer. In this view, several studies investigated tumor samples for the presence of various viral DNA with conflicting results.

**Findings:**

We undertook a systematic DNA analysis of 44 consecutive, prospectively collected primary tumor samples by real time and qualitative PCR for viruses of known or potential oncogenic role in humans, including polyomavirus (JCV, BKV, Merkel cell polyomavirus), HPV, HTLV, HHV-8 and EBV. Negative controls consisted of surgical resection margins. No evidence of genomic DNA fragments from tested virus were detected, except for EBV, which was found in a significant portion of tumors (23/44, 52%). Real-time PCR showed that EBV DNA was present at a highly variable content (median 258 copies in 10^5^ cells, range 15–4837). Presence of EBV DNA had a trend to be associated with high lymphocyte infiltration (p = 0.06, *χ*2 test), and in situ hybridization with EBER1-2 probes revealed latency in a fraction of these lymphoid cells, with just a few scattered plasma cells positive for BZLF-1, an immediate early protein expressed during lytic replication. LMP-1 expression was undetectable by immunohistochemistry.

**Conclusions:**

These results argue against a significant involvement of the tested oncogenic viruses in established colon cancer.

## Findings

It is estimated that 16-18% of the global cancer burden can be associated with oncogenic viruses [1,2]. The DNA viruses of recognized pathogenic role in humans include HCV, HBV, HPV, HHV-8, MCPyV and EBV, while the role of polyomaviruses JCV and BKV is controversial [1,3]. Colon cancer is a leading cause of cancer-related death and morbidity in western countries. The pathogenesis of this cancer is highly complex and it involves sequential genetic and epigenetic mechanisms [4,5]. A possible contribution of environmental agents, including bacteria and viruses, is also considered [6,7]. However, the search for a pathogenic agent generated conflicting results, possibly related to technical reasons or other unknown factors [6].

**Figure 1:**
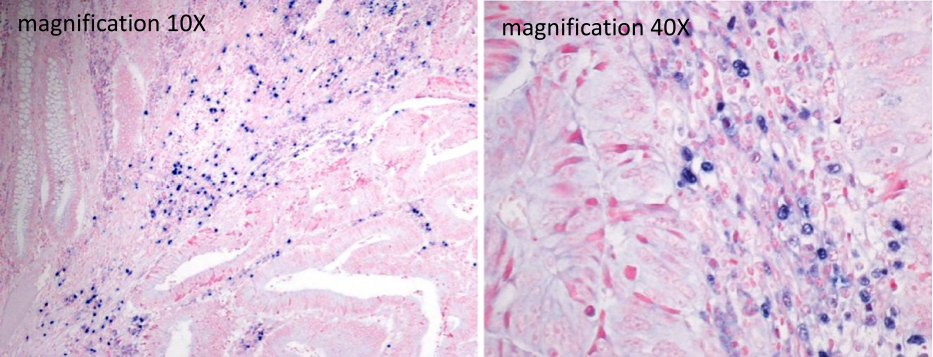
**EBV latency is restricted to the inflammatory infiltrate in colon cancer.** Tissue RNA in situ hybridization using EBER 1–2 PNA probes to detect EBV latency (Dako, Denmark). A blue nuclear staining indicates a positive reaction. Epithelial and cancer cells are negative, whereas occasionally positivity of non-neoplastic lymphoid cells may be extensive (reaching 20%).

**Figure 2:**
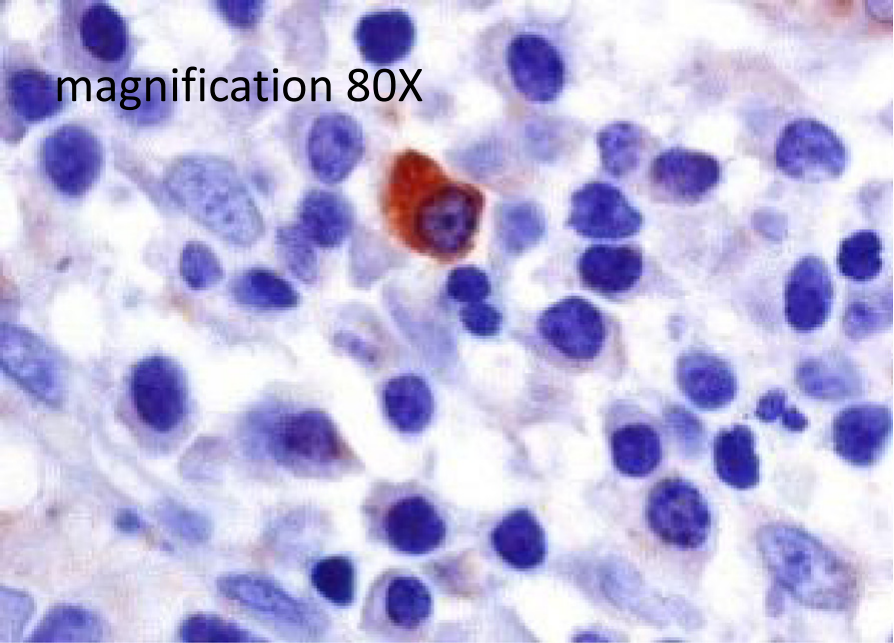
**EBV lytic phase was limited to a few scatter plasma cells in the lymphoid infiltrate.** Immunohistochemistry with a monoclonal antibody specific for the early immediate protein BZLF-1. These findings confirmed that EBV is essentially in latency phase in the lymphoid infiltrates of colorectal cancers.

To contribute to this issue, we prospectively collected a series of consecutive 44 colon cancers to perform a systematic PCR-analysis for human polyomaviruses (JCV, BKV, MCPyV), HPV, HTLV, HHV-8 and EBV. The study was approved by Fondazione IRCCS Policlinico San Matteo Review Board. Fondazione IRCCS Policlinico San Matteo Review Board. Clinical and pathology characteristics are reported in Table 1. The population was representative of colon cancer cases who underwent primary tumor resection (low numbers of metastatic cases, Duke’s stage D). Internal control consisted of surgical resection margin (at least 5 cm distant from the tumor). Quality of extracted DNA samples (QIAmp DNA Mini Kit, Qiagen, Italy) was verified by means of amplification of the housekeeping gene beta-2-microglobulin (DNA amplicons >10^5^ copies). Positive controls included virus infected human samples from various pathologies, including tumor samples, and plasmid DNA. Amplification methods consisted of real time (EBV, JCV, BKV, HTLV and HHV-8) [8-11] and qualitative PCR (MCPyV, HPV) [12,13]. Given the very controversial evidence concerning JCV, which was found from 0% to nearly 80% of the tumor samples tested in the literature [6,14-18], JCV was additionally investigated by the specific qualitative PCR described in positive reports [18] and that employed primers spanning a different portion of the large T antigen. Sensitivity for JCV real-time PCR assay was 1 viral copy in 10^5^ cells. Experiments showed that no genomic DNA fragments from the tested viruses were detectable, with the notable exception of EBV that was positive in a consistent portion of cases (23/44 samples, 52%). Tumors associated with EBV positivity had EBV negative surgical resection margins. EBV DNA content was highly variable in tumors (median 258 viral copies in 10^5^ cells, range 15–4837), and EBV had a trend to be observed in tumors displaying high lymphoid infiltration (p = 0.06, *χ*^2^ test). In situ hybridization analyses for the detection of EBER1-2 RNAs (PNA Probe/FITC and ISH detection kit, Dako, Denmark) demonstrated virus latency in a variable fraction of infiltrating non-neoplastic lymphoid cells, which could reach 20% in a few cases (Figure 1), but not in tumor cells. On the same line, immunohistochemistry to EBNA-1 nuclear protein (Fitzgerald Industries International – USA, clone M5042521), an antigen that is expressed during both latent and lytic phases, failed to show positive nuclei in neoplastic cells. Immunohistochemistry for LMP-1, a membrane associated protein involved in activation, was also negative, while lytic cycle was detected via expression of the immediate early protein BZLF1 (ZEBRA antigen, LSBio, clone LS-C102904) in a few scattered plasma cells (Figure 2). These findings essentially confirm latency of EBV in lymphoid infiltrates. The presence of EBV was not associated with the other tumor or clinical parameters studied including age, stage, tumor localization, or the presence of necrosis.

**Table 1:** Clinical and pathological features of the colon cancer cases analysed for potential oncogenic viruses

**Colon cancer (n = 44)**	**No. of cases**	**%**
Sex		
Male	26	59
Female	18	41
Age groups		
Median of age = 75 years (range 46–84)		
≤ 65 years	9	20
≥ 65 years	35	80
Anatomic site		
Caecum	4	9
Ascending	11	25
Transverse	9	20
Descending	6	14
Sigmoid	4	9
Sigmoid - Rectal	10	23
Colon dx	18	41
< 65 years	26	59
Staging		
B1	7	16
B2	16	36
C1	2	5
C2	15	34
D	4	9
TNM		
T1	0	0
T2	9	20
T3	29	66
T4	6	14
N0	23	52
N1	11	25
N2	10	23
M0	40	91
M1	4	9
Grading		
G1	0	0
G2	32	73
G3	12	27

In conclusion, we performed a PCR-based systematic analysis for potential oncogenic viruses in clinically established colon cancer and EBV was the only one detected. The viral infection was restricted to latency in the lymphoid infiltrate, in line with the few reports that used in situ hybridization with EBER probes [19,20], while we noted an association with high lymphocyte infiltration, a well-recognized favorable prognostic parameter. EBV positivity in lymphoid infiltrates may occasionally be extensive (Figure 1), much higher than expected on the numbers of circulating EBV positive memory B-lymphocytes in normal individuals, and it might be of interest to studying this phenomenon in specifically designed studies. In summary, the present analysis does not support a significant involvement of the tested viruses in manifest colon cancer, and suggests that new approaches [21] capable of detecting known and unknown non-human sequences should be investigated to study the role of infectious agents in colon cancer.

## Competing interests

The authors declare that they have no competing interests.

## Authors’ contributions

VP and FB designed and coordinated the study and wrote the manuscript; MR collected data on patients and contributed to elaboration and interpretation of results; LF and SP performed PCR experiments; ED and RR carried out in situ hybridization and immunohistochemistry analyses; OL performed histological evaluation; AV performed primary tumor collection, histological evaluation and contributed to study design and interpretation of results, reviewed the manuscript; SB collected data on patients and critically reviewed the manuscript; MP critically reviewed the manuscript; PP contributed to study coordination and critically reviewed the manuscript. All authors read and approved the final manuscript.
